# On Hemorrhage in Fevers

**Published:** 1856-03

**Authors:** Henry Kennedy

**Affiliations:** Fellow of the King and Queen’s College of Physicians in Ireland, &c.


*On Hemorrhage in Fevers. Extract from an Essay.* By HENRY KENNEDY, A. B., M. B. T. C. D., Fellow of the King and Queen's College of Physicians in Ireland, &c.

Before concluding this essay, it may probably be expected that something should be said on the treatment of fevers attended with hemorrhage. Were I, however, to enter fully into this part of my subject, it would extend the essay beyond all reasonable limits; and I shall, therefore, confine myself to a few remarks of the most general character.

The preceding pages illustrate—I would hope amply—the fact that hemorrhages in one form or other, are of frequent occurrence in the fevers of Dublin; and farther, that these hemorrhages are, in the great majority of instances, beneficial. The contemplation of these facts appears of the greatest moment. For it will be observed they are the doings of Nature, not of Art. They constitute a part, so to speak, of the natural progress of our fevers, and go on quite independent of interference on our side. It must be repeated, too, that, speaking generally, their effects are beneficial, often in the most marked way, of which many examples have been given in the preceding pages. Now, with such facts before us, what is the direct corollary which follows from them? Can any reasonable deduction be made, which will tend to improve our treatment of these diseases? Clearly, that it is the part of the physician to imitate, it may be, or at least to second, the efforts of Nature; and that every endeavor should be made to attain that knowledge which will, at least, not thwart her. Hence follows the great and leading principle which I believe should guide us in by far the greatest number of cases of fever in which hemorrhage occurs—that of non-interference: I mean an interference which would tend to check the hemorrhage directly. That cases do occur where we must step in, and do our best to check bleeding, is certain. But such are the exceptional cases, not the rule.

In many instances, however, we are fully justified in acting so as to assist the bleeding. Thus, epistaxis may be encouraged, and often with the best result. Or again, cases are very common where but a few drops of blood may be lost from the nose; and here, by taking the hint, we can have no hesitation in the application of some leeches, proportioned in number to the demands of each individual case. I have never yet seen an instance where Nature led the way, and Art then came to her assistance, that the relief afforded was not of the most marked kind; and it is really curious what a small number of leeches will often answer the purpose; even so few as two, and to an adult, I have repeatedly ordered with advantage. These may have to be repeated, but it is the general principle only I am speaking of here.

There is another mode in which a kind of interference, in cases of epistaxis, of which I am now speaking, proves most salutary. I mean the use of aperients. It will often be found on inquiry that a confined state of the bowels is keeping up a bleeding, which it may be thought necessary to moderate. In such cases, aperients act often like magic, lessening not only the headache, which is so apt to attend such a state, but moderating also the bleeding, if not checking it entirely. In these ways, then, whilst we still keep to the rule of no direct interference, that is, in the great majority of instances, Art may step indirectly in, and be of the most essential service.

The remarks just made apply mainly to the first week of fever, though they may, with a proper discretion, be used later. As the disease goes on, however, and bleeding from the nose occurs at later stages of fever, there is then less occasion for interference. The amount of blood lost is generally, at this period, very small, and puts on more of the passive character. In such it is best met by wine and other stimulants; and this treatment is still more called for when bleeding occurs, as it may do, during convalescence.

Hemorrhage from the chest is to be treated on the same principles as those indicated for epistaxis. Dry cupping is worthy of more general use than it receives; and, in suitable cases, blood may be taken in this way with marked advantage. Blisters, too, I have seen of use. The same remarks apply to bleeding, which is in direct connexion with pneumonia, of which instances have been already given.

Hemorrhages from the bowels, occurring in fever, require more consideration than any of those just glanced at. For here the loss of blood is often very considerable, so much so, as to affect seriously the vital functions; and, if allowed to go on, destroy life itself. Instances of this kind have been already given. In such there is nothing for it but endeavor to check it by all the means in our power. The medicines which appear to me most useful are the gallic acid and the oil of turpentine; either with or without a small quantity of opium or laudanum. I think I have seen decided benefit from both but especially from the latter. It may be given in ten-drop doses, frequently repeated, and in the form of emulsion. In those instances where the urgency is not so great, yet where the tendency to bleeding still continues, I have seen the turpentine, combined with caster-oil, act like a charm. Dr. George Kennedy is in the constant habit of using it in this way. One or two drachms of each, according to circumstances, are given in draught. Injections have not appeared to me as useful as might be expected, possibly because they do not reach that part of the bowel where the bleeding comes from.

Of uterine hemorrhages little need be said. If miscarriage be threatened, I believe it is next to useless to attempt to stop it. I have not witnessed any case where, when once threatened, it did not go on. I speak now of the earlier months of pregnancy; at a more advanced stage, however, as for instance at six or seven months, I have seen cases of threatened miscarriage, and yet it did not occur. If the bleeding be a pure hemorrhage, it will be treated on the same principles as bleeding from any other part of the frame, and of course only interfered with directly when it is to such an extent as to affect the vital functions. Lastly, if the bleeding be the natural function, it will not be interfered with, to check it, but may be encouraged with marked advantage.

To sum up, then, the principles which I believe should guide us when hemorrhage occurs in fevers, the following may be stated:—

1. That direct interference is the exception, not the rule.
2. That indirect interference, as by the means already alluded to, may be, and often is, most judicious and beneficial.
3. That direct interference is only justified where the hemorrhage is to such an extent as to affect the vital functions.—*Dublin Quarterly Journal.*

